# Advanced superhard composite materials with extremely improved mechanical strength by interfacial segregation of dilute dopants

**DOI:** 10.1038/s41598-020-78064-0

**Published:** 2020-12-03

**Authors:** Tomohiro Nishi, Katsuyuki Matsunaga, Takeshi Mitsuoka, Yasuyuki Okimura, Yusuke Katsu

**Affiliations:** 1 Department of Materials Physics, Nagoya University, Furo-cho, Chikusa-ku, Nagoya, 464-8603 Japan; 2 NGK Spark Plug Co., Ltd., Komaki-shi, Iwasaki, Aichi 485-8510 Japan; 3 Nanostructures Research Laboratory, Japan Fine Ceramics Center, 2-4-1, Atsuta-ku, Mutsuno, Nagoya, 456-8587 Japan

**Keywords:** Materials science, Structural materials, Composites, Engineering, Mechanical engineering

## Abstract

Control of heterointerfaces in advanced composite materials is of scientific and industrial importance, because their interfacial structures and properties often determine overall performance and reliability of the materials. Here distinct improvement of mechanical properties of alumina-matrix tungsten-carbide composites, which is expected for cutting-tool application for aerospace industries, is achieved via interfacial atomic segregation. It is found that only a small amount of Zr addition is unexpectedly effective to significantly increase their mechanical properties, and especially their bending strength reaches values far beyond those of conventional superhard composite materials. Atomic-resolution STEM observations show that doped Zr atoms are preferentially located only at interfaces between Al_2_O_3_ and WC grains, forming atomic segregation layers. DFT calculations indicate favorable thermodynamic stability of the interfacial Zr segregation due to structural transition at the interface. Moreover, theoretical works of separation demonstrate remarkable increase in interfacial strength through the interfacial structural transition, which strongly supports reinforcement of the interfaces by single-layer Zr segregation.

## Introduction

Monolithic metal-oxide ceramics have high refractory capabilities and good chemical stability, and are expected for structural applications in harsh operating conditions such as high stresses and high temperatures. However, they intrinsically have brittleness and show catastrophic fracture behavior at low temperatures, which degrades their reliability and thus hampers their practical structural applications. In order to overcome such drawbacks, ceramic matrix composites (CMCs) were developed, and can have improved strength and fracture toughness depending on choices of secondary phases^1–3^. Therefore, CMCs are recently of growing importance in aerospace and power-generation industries for use as parts of jet engines and gas turbines^4,5^. Cutting tools for nickel super alloys (substances for the jet engines and gas turbines) are now being another important application of CMCs, in response to strong demands from industries for processing parts of the engines and turbines quickly, efficiently, and economically^6^. SiC-whisker reinforced Al_2_O_3_ is currently most commonly used for cutting tools, which has more superior chemical stability, wear resistance, and hardness than another candidate of tungsten carbide-cobalt (WC–Co) cemented carbide^7^. However, SiC whiskers are generally very expensive, and careful handling is also required in processing, in order to avoid potential health hazards by SiC whiskers having a needle-like shape. Moreover, as will be shown below, mechanical strength of SiC-whisker reinforced Al_2_O_3_ is limited to at most 1.2 GPa. With forthcoming prosperity of aerospace industries, it is more desirable to obtain CMCs exhibiting improved strength and hardness at lower cost as well as without any risk for health, which can offer longer life times and higher metalworking speeds to cutting tools.

**Figure 1:**
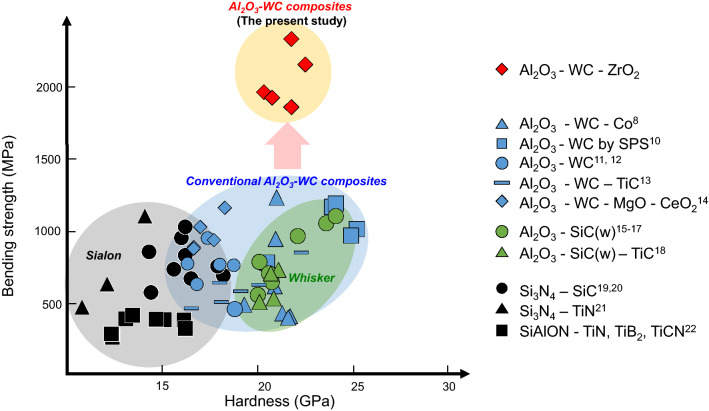
Bending strength versus hardness for various CMCs. Al_2_O_3_-WC composites developed in the present study achieved the bending strength of more than 2 GPa, which is much larger than those of conventional CMCs.

In this study, Al_2_O_3_ matrix tungsten carbide (Al_2_O_3_-WC) are found out to have distinct mechanical properties that are much superior to conventional CMCs. Al_2_O_3_ is a typical material for structural application because of its high hardness and chemical inertness, while WC is often used as a superhard material. As a matter of fact, a combination of Al_2_O_3_ and WC is expected as a potential candidate for cutting-tool materials. For this purpose, a number of specific powder synthesis and sintering techniques such as vacuum hot pressing and spark plasma sintering (SPS) were applied to Al_2_O_3_-WC so far^8,9^. In spite of the long history and extensive studies of CMCs, bending strengths of around 1 GPa for Al_2_O_3_-WC were attained at best, and still cannot excel performance of conventional CMCs (see Fig. 1)^8,10–22^.

The present study selects small amount of ZrO_2_ as an additive, and succeeds in producing Al_2_O_3_-WC with as large a bending strength as more than 2 GPa, which is far beyond those of conventional CMCs for their cutting-tool applications. Microstructures and interfaces in the composites are characterized by X-ray diffraction (XRD), scanning electron microscopy (SEM), and scanning transmission electron microscopy (STEM). The presence of specific interfacial segregation behavior of Zr atoms in a single atomic layer between Al_2_O_3_ and WC grains is observed, which is most likely to reinforce the interfaces significantly. Density functional theory (DFT) calculations are used to reveal an effect of Zr segregation for the interfaces, and determine an atomic-level mechanism for interface strengthening by single-layer Zr addition.

## Materials and methods

### Materials preparation

Starting materials were α-Al_2_O_3_ powders with an average grain size of approximately 0.5 µm and 99.9% purity (RC-HP-DBM, Baikowski-Malakoff Inc.), and WC powders with an average grain size of 0.7 µm and 99.8% purity (WC04NR, A.L.M.T. Corp.), and ZrO_2_ powders with an average grain size of 1.5 µm and 99.8% purity (EP, Daiichikigenso Kagakukogyo CO. Ltd.). After weighing the respective powders, preliminary pulverization was performed. In the process of preliminary pulverization, Al_2_O_3_ and ZrO_2_ powders were ball milled for about 20 h with an ethanol solvent using high purity alumina balls as grinding media. In order to obtain dispersed slurries for the composites, WC powders, a solvent and a dispersant were added to powder mixtures of Al_2_O_3_ and ZrO_2_ in the ball mill and further mixing and crushing were conducted for about 20 h. By heating the slurries in a water bath, solvents were removed from the slurries. The powder mixtures sieved with a mesh size of 250 µm and a wire diameter of 160 µm were loaded into the 31 × 31 mm square graphite die. Specimens were then sintered via a hot-pressing technique in a flowing Ar atmosphere at 1750 °C for 2 h under a fixed uniaxial pressure 30 MPa. The resultant sintered composites were found to have relative densities of more than 99%, which were measured with the Archimedes method in deionized water, indicating fully sintered bodies of the specimens.

### Mechanical property measurement

Sintered specimens were cut for mechanical tests and their surfaces were polished using diamond pastes. Specimens for mechanical tests were formed in a shape of a prism having a rectangular cross section and with dimensions of 15 × 4 × 3 mm^3^. Bending strengths were measured using a three-point bending technique with a loading span length of 10 mm and a cross-head speed of 0.5 mm min^−1^. Three-point bending always gives artificially high strengths compared to four-point bending because of the small volume under high load. The bending strength measured by four-point bending technique of Al_2_O_3_ prepared by hot pressing in the past was 588 MPa^23^, which was approximately 10% lower than that of this work. Hardnesses were measured with a Vickers hardness tester with a load of 9.8 N load and a holding time of 15 s, according to JIS R 1610^24^. Fracture toughnesses were determined by the indentation fracture (IF) method, according to JIS R 1607^25^.

### Cutting experiments

Cutting tools manufactured from the hot-pressed composites had a shape specified by a code “RCGX120700T01020” in conformity with JIS B 4210. Materials metalworked were cast products made of Inconel 718 with a pierced disk shape 250 mm in diameter. Cutting tests were carried out at cutting speeds of 240, 360 and 480 m min^−1^ in wet machining.

### X-ray diffraction (XRD) measurement

XRD data were obtained on an automated multipurpose X-ray diffractometer (Smart Lab, Rigaku) operated at 40 kV and 30 mA using Cu-Kα radiation covering 25°–55° (2*θ*) with a scanning step of 0.02°. The diffraction data were collected at room temperature.

### Scanning electron microscopy (SEM) observation

SEM images were acquired with a field-emission SEM (JSM-7001F, JEOL Ltd.) operated at an accelerating voltage of 5 kV.

### TEM specimen preparation

Specimens for TEM observation were obtained using mechanical grinding to a thickness of about 50 µm, dimpling to a thickness of about 10 µm, and finally argon ion beam milling (Gatan PIPS-II model695) to electron transparency.

### Scanning transmission electron microscopy (STEM) observation

HAADF and ABF STEM observations were performed using an aberration-corrected STEM (JEM-2100F, JEOL Ltd.) at an accelerated voltage of 200 kV with spherical aberration coefficient of ~ 0.5 mm giving an optimum probe-size of ~ 1.0 Å. A high-angle angular dark-field (HAADF) detector with an inner angle greater than 73 mrad was used. EDS were performed using a STEM (HD-2000, HITACHI) at an accelerated voltage of 200 kV with probe-seize of 2.4 Å.

### Density functional theory (DFT) calculation

DFT calculations based on the projector augmented wave method in the VASP program were used in the present study^26^. The generalized gradient approximation (GGA) parameterized by Perdew, Burke and Ernzerhof was used for exchange–correlation interactions^27^. Wavefunctions were described by plain waves up to a cutoff energy of 400 eV. Brillion zone integration was performed only at a Γ point for supercells of the interfaces described below. Structural optimization was truncated when residual forces on all atoms became less than 0.01 eV Å^−1^.

In order to analyze electronic and atomic structures of interfaces between α-Al_2_O_3_ and WC, supercells containing α-Al_2_O_3_ and WC slabs were generated. An orientation relationship between the two slabs was determined to be (0001)_Al2O3_||(0001)_WC_ and $[10\bar{1}0]_{{{\text{Al}}2{\text{O}}3}} ||[11\bar{2}0]_{{{\text{WC}}}}$. This is because previous EBSD measurements showed that this is one of two orientation relationships with the highest observation frequencies in the Al_2_O_3_-WC composites^28^. As shown in Supplementary Fig. 1, however, the present orientation relationship results in large lattice misfits of 3.9% for the $[10\bar{1}0]_{{{\text{Al}}2{\text{O}}3}}$ direction (the $[11\bar{2}0]_{{{\text{WC}}}}$ direction) and 65% for the $[11\bar{2}0]_{{{\text{Al}}2{\text{O}}3}}$ direction (the $[10\bar{1}0]_{{{\text{WC}}}}$ direction). Therefore, the minimum unit of WC(0001) toward $[10\bar{1}0]_{{{\text{WC}}}}$ was extended by three times so as to make the lattice misfit minimized. The resultant lattice misfit of the extended supercell in the $[10\bar{1}0]_{{{\text{WC}}}}$ direction was 3.9%. Subsequently, initial supercell edge lengths parallel to the interface plane were adjusted to intermediate values of periodic lengths of the Al_2_O_3_ slab and the extended WC slabs, so as to impose equal amounts of lateral elastic strains on the two slabs. Supercell edge lengths were also relaxed by structural optimization.

Supercells used in this study involved alternative stacking of two Al_2_O_3_ and two extended WC slabs. This is because α-Al_2_O_3_ does not have an inversion symmetry along the <0001> direction, unlike hexagonal WC. Two Al_2_O_3_ slabs with the opposite [0001] directions were arranged toward the *c* axis of the supercell, and were separated by the extended WC(0001) slabs. This can offer symmetric interfaces at both ends of each WC(0001) slab. Thicknesses of Al_2_O_3_ (0001) and WC(0001) slabs were taken to be more than 1 nm, so as to prevent spurious interactions between the interfaces over the repeated supercells. The resultant total number of atoms in the supercell was 588. In calculations of Zr segregation, Zr atoms were introduced into all interfaces of the supercells in a similar manner. The structure models were illustrated with the VESTA program^29^.

Defect formation energies were evaluated from total energies of the interface supercells. In the case of a substitutional Zr atom at a particular cation site M (with an effective charge *q*), the formation energy ${\Delta E}_{f}^{\mathrm{Zr}}$ can be obtained as follows.

1 $${\Delta E}_{f}^{\mathrm{Zr}}={E}_{T}\left(\mathrm{doped}\right)-{E}_{T}\left(\mathrm{undoped}\right)+\sum_{\mathrm{i}}{\mu }_{\mathrm{i}}+q\left({E}_{\mathrm{VBM}}+{\varepsilon }_{F}\right).$$

Here *E*_*T*_(doped) and *E*_*T*_(undoped) are total energies of interface supercells with and without substitutional Zr, respectively. *μ*_*i*_ is an atomic chemical potential of atomic species i, which was determined by assuming chemical equilibrium among Al_2_O_3_, WC, ZrO_2_, and ZrC. A choice of solid ZrC in the chemical equilibrium assumption can be rationalized from the fact that the ZrC phase can be formed under hot pressing at high temperatures^11^.

When substitutional Zr^4+^ occupies an Al^3+^ site, for instance, its effective charge is equal to +1 and then its formation energy depends on the Fermi energy *ε*_F_. Since *E*_VBM_ of the defective supercell is generally different from that in the perfect supercell, *E*_VBM_ of the defective supercell should be determined. In this study, *E*_VBM_ corrections using average electrostatic potentials were made^30,31^. Moreover, it is assumed that substitutional Zr^4+^ at an Al^3+^ site accompanies Al^3+^ vacancies as charge compensating defects, according to the following defect reaction of ${\mathrm{ZrO}}_{2}\to {\mathrm{Zr}}_{\mathrm{Al}}^{\cdot }+\frac{1}{3}{\mathrm{V}}_{\mathrm{Al}}^{\mathrm{^{\prime}}\mathrm{^{\prime}}\mathrm{^{\prime}}}+2{\mathrm{O}}_{\mathrm{O}}^{\times }$, where the Kröger-Vink notation was used. Then, a substitutional Zr^4+^ defect and an Al^3+^ vacancy was introduced at a particular Al^3+^ site in separate interface supercells, and their individual formation energies ($\Delta {E}_{f}({\mathrm{Zr}}_{\mathrm{Al}}^{+})$ and $\Delta {E}_{f}({\mathrm{V}}_{\mathrm{Al}}^{3-})$, respectively) were used to obtain ${\Delta E}_{f}^{\mathrm{Zr}}$ as follows:

2 $${\Delta E}_{f}^{\mathrm{Zr}}=\frac{1}{4}\left\{3\Delta {E}_{f}({\mathrm{Zr}}_{\mathrm{Al}}^{+})+\Delta {E}_{f}({\mathrm{V}}_{\mathrm{Al}}^{3-})\right\}$$

This postulates that charge compensating Al^3+^ vacancies do not explicitly interact with substitutional Zr^4+^ defects around the interface although they are located at equivalent Al^3+^ sites.

A work of separation at the interface (*W*_*s*_) represents ideal strength of the interface. This was obtained from a total energy of the interface supercell minus those of the Al_2_O_3_ and WC surface slabs divided by an interface area. The surface slabs were generated from the interface supercell, by removing either Al_2_O_3_ or WC slabs and making vacuum areas. In this case, total energies for the surface slabs were calculated after structural optimization.

## Results and discussion

### Mechanical properties of Al_2_O_3_-WC-ZrO_2_ composites

**Figure 2:**
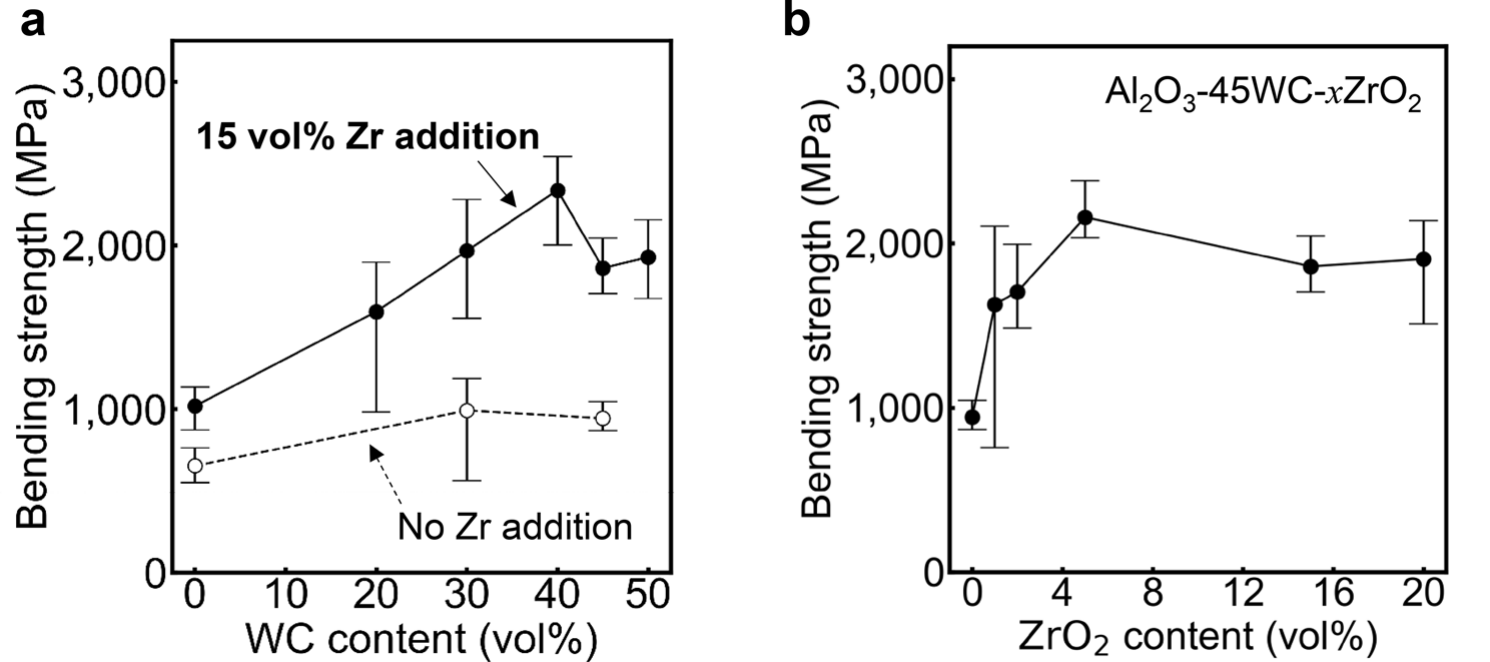
Bending strengths of Al_2_O_3_-WC composites. (**a**) bending strength against WC content for Al_2_O_3_-WC-15ZrO_2_ composites. (**b**) bending strength against ZrO_2_ content *x* for Al_2_O_3_-45WC-*x*ZrO_2_ composites.

Figure 2a shows measured bending strengths of Al_2_O_3_-WC composites hot pressed at 1750 °C for 2 h in Ar atmosphere. It is generally considered that bending strength of composites is sensitive to mechanical strength of grain boundaries and interfaces in the microstructures^1,32,33^. Weak interfacial bonding can be suspected for Al_2_O_3_-WC because Al_2_O_3_ and WC grains are intrinsically difficult to undergo sintering. When microstructures of the present composites after fracture were observed (see Supplementary Fig. 2), it was confirmed that cracks are often deflected at interfaces between Al_2_O_3_ and WC. Interfacial crack deflection can generally contribute to increased fracture toughness, which can also be confirmed from the fact that fracture toughness of the composites increases with increasing WC contents (also see Supplementary Fig. 3). However, bending strengths of the composites without ZrO_2_ addition do not show explicit increase with rising WC contents, which may indicate weak interfacial bonding strength between Al_2_O_3_ and WC grains. Moreover, bending strengths around 1 GPa for the Al_2_O_3_-WC composites with 45 vol% of WC were also as larger as those of other composites such as SiC-whisker reinforced Al_2_O_3_ and SiAlON-TiN reported previously (see Fig. 1)^15–22^. Since bending strengths are closely related to life times and cutting speeds of tools, which should be further increased for their practical use. In contrast, Al_2_O_3_-WC composites with some amounts of ZrO_2_ show improved bending strengths. When 15 vol% of ZrO_2_ is added into the composites, for instance, their bending strengths tend to largely increase with increasing WC contents. It is noted that the bending strengths as a function of WC content once decrease at 40% WC. However, the strengths again start to rise for further WC contents, eventually approaching to those of pure WC (see Supplementary Fig. 4). It is worth mentioning that the bending strengths at around 40 vol% of WC exceeds 2 GPa, which is more than twice larger than values of Al_2_O_3_-WC without ZrO_2_ addition or other conventional SiC-whisker reinforced composites (Fig. 1). ZrO_2_ addition does not degrade Vickers hardnesses and fracture toughnesses but increases them with rising WC contents (Supplementary Figs. 3a and 3b). Therefore, ZrO_2_ addition is substantially effective for Al_2_O_3_-WC to improve their mechanical properties.

The bending strengths for composites with 45 vol% WC as a function of ZrO_2_ content *x* (denoted as Al_2_O_3_-45WC-*x*ZrO_2_ hereafter) are displayed in Fig. 2b. Variations of Vickers hardnesses, fracture toughnesses for Al_2_O_3_-45WC-*x*ZrO_2_ are displayed in Supplementary Fig. 3c, d. It is obvious that bending strengths are more strongly affected by ZrO_2_ contents, as compared with hardnesses and fracture toughnesses. Bending strengths of the composites rapidly rise up to 5 vol% of ZrO_2_, and then are leveled off at around 2 GPa for more ZrO_2_ contents. This tendency indicates that larger ZrO_2_ amounts, which would result in formation of secondary ZrO_2_ phases in the microstructures, do not directly contribute to improvement of the bending strength.

It can be seen in Supplementary Figs. 5a and 5b that a SEM micrograph of the sample with *x* = 5 vol% is quite similar with the one without ZrO_2_ addition (*x* = 0 vol%). In addition, it is confirmed from Supplementary Figs. 5a and 5b that the size distributions of interconnected skeletons of WC grains are not changed by the addition of ZrO_2_. XRD patterns for the composites in Supplementary Fig. 6 demonstrate that peaks coming from the presence of secondary ZrO_2_ phase can be observed for *x* = 15 vol% whereas explicit peaks from ZrO_2_ or other Zr-related phases are absent in the profile for the composite of *x* = 5 vol%. Therefore, ZrO_2_ does not reside as any secondary-phase grains in the system with *x* = 5 vol%. On the other hand, Zr solubility in Al_2_O_3_ and WC grains is negligibly small^34,35^, so that added ZrO_2_ could dissolve into interfacial regions in the microstructure. Even if the secondary ZrO_2_ phase is absent, the bending strength can be highly enhanced by only a small amount of ZrO_2_ addition.

### Effect of ZrO_2_ addition

**Figure 3:**
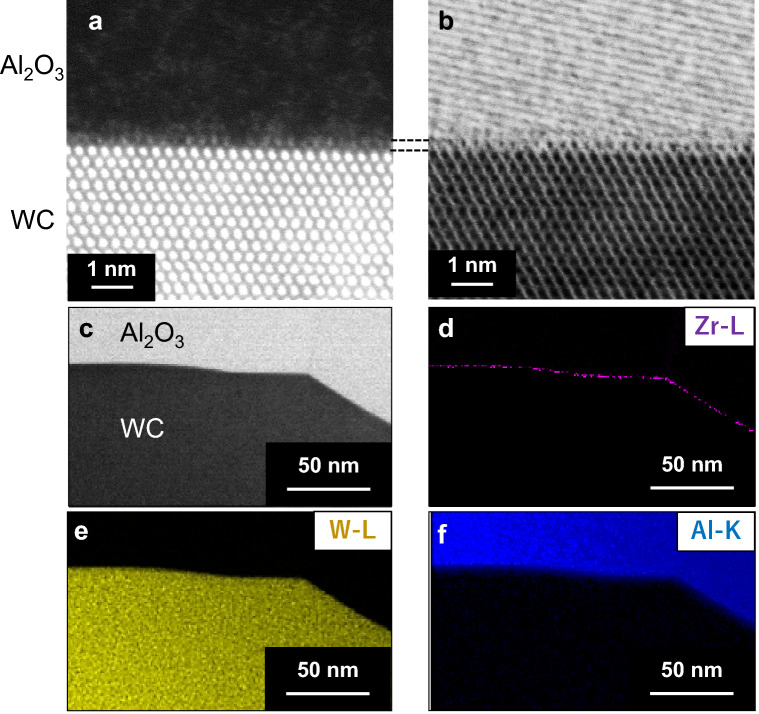
HAADF and ABF STEM images and STEM-EDS for specimens of Al_2_O_3_-45WC-5ZrO_2_. (**a**) HAADF-STEM image and (**b**) ABF-STEM image for an interface between Al_2_O_3_ and WC grains of Al_2_O_3_-45WC-5ZrO_2_. (**c**–**f**) STEM image and corresponding element mapping images.

In order to reveal a ZrO_2_ effect for the mechanical properties, STEM observations are performed for specimens of Al_2_O_3_-45WC-5ZrO_2_. Figure 3a,b show typical high angle annular dark field (HAADF) and annular bright field (ABF) images for an interface between Al_2_O_3_ and WC grains. Owing to image intensities proportional to atomic numbers (Z) in the HAADF image, atomic columns in WC grains look much brighter than those in Al_2_O_3_. In contrast to the Z-dependent HAADF image, it is known that the ABF image can provide brighter intensities for atomic columns containing lighter elements, so that atomic columns in Al_2_O_3_ can be observed more brightly than those in WC in the ABF image. As can be seen, the image contrast at the interface layer about 0.3 nm in thickness in the ABF image shows some resemblance to that in WC. It is worth claiming that the corresponding interfacial atomic layer in the HAADF image involves relatively weak but explicit bright spots. Since the specimen involves ZrO_2_ addition, such interfacial bright spots on the WC grain may be related to Zr atoms segregating at the interface. This speculation can be rationalized from nano-probe energy dispersive spectroscopy (EDS) element mapping in the STEM. It is clear that a continuous Zr segregation layer is formed along the interface between Al_2_O_3_ and WC grains (Fig. 3c–f). From STEM observations for a number of different areas in the microstructures, it was also confirmed that such Zr segregation layers are formed at almost all the interfaces even in the samples with 5% ZrO_2_ addition, which is closely related to the saturated bending strengths of the composites over 5% ZrO_2_ addition (see Fig. 2b). Therefore, the bright spots in the single atomic layer on the WC grain in the HAADF image is an indication that Zr atoms segregate at the interface by replacing a number of W atoms on the outermost atomic layer of WC, forming atomic columns mixed with W and Zr atoms along the incident beam direction. Since intense Zr segregation was not explicitly observed at the Al_2_O_3_ grain boundaries (shown in Fig. 3c–f) as well as WC grain boundaries (Supplementary Fig. 7), the doped Zr atoms appear to be preferentially located at the interface between Al_2_O_3_ and WC particles, forming an atomic segregation layer. This is a representative feature of Zr segregation through the microstructures of the composites. It should be noted that XRD and STEM observations of Al_2_O_3_-45WC-5ZrO_2_ with bending strength of more than 2 GPa do not detect the presence of secondary phases or nanoparticles containing Zr (Supplementary Fig. 6). Even if Zr ions segregate at Al_2_O_3_ grain boundaries, an effect of Zr segregation on Al_2_O_3_ grain boundaries should be limited because the bending strength of Al_2_O_3_ polycrystals increases by only about 350 MPa with Zr addition as shown in Fig. 2a (WC content = 0%). Therefore, it is speculated that the Zr segregation at the Al_2_O_3_-WC interface is a major factor for the measured significant improvement of mechanical properties in the Al_2_O_3_-WC composites.

In order to reveal an effect of Zr segregation at interfaces between Al_2_O_3_ and WC in more detail, DFT calculations are performed with slab models. Although real composite materials contain a range of interfaces, it is essential to consider which orientation relationship between the two adjacent crystals is preferred in Al_2_O_3_-WC. In this regard, electron back scattering diffraction (EBSD) studies for Al_2_O_3_-WC demonstrated that (0001)_Al2O3_||(0001)_WC_ and $[10\bar{1}0]_{{{\text{Al}}2{\text{O}}3}} ||[11\bar{2}0]_{{{\text{WC}}}}$ is one of the two orientation relationships showing the highest frequencies, which is considered in the present study. However, this orientation relationship still involves large lattice misfits of 3.9% for the [10_10]_Al2O3_ direction and 65% for the $[11\bar{2}0]_{{{\text{Al}}2{\text{O}}3}}$ direction (see Supplementary Fig. 1). Therefore, the periodic WC unit along the $[10\bar{1}0]_{{{\text{WC}}}}$ direction are triply extended so as to make the lattice misfit of the extended slab model toward the $[11\bar{2}0]_{{{\text{Al}}2{\text{O}}3}}$ direction (the $[10\bar{1}0]_{{{\text{WC}}}}$ direction) minimized from 65 to 3.9%.

**Figure 4:**
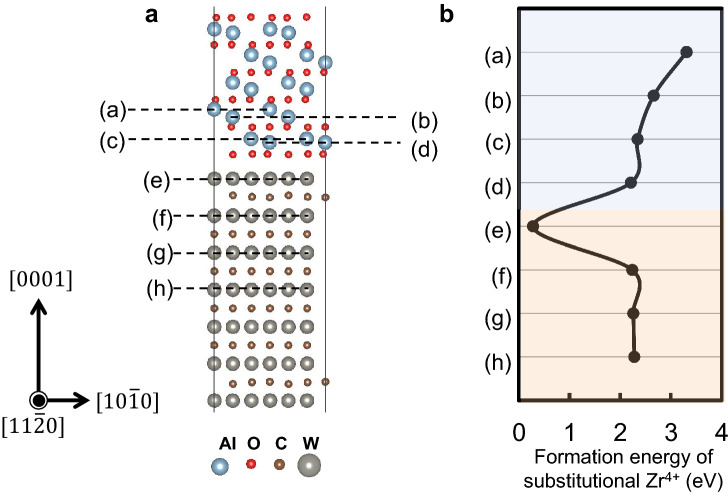
Most stable atomic structure for Al_2_O_3_(0001)/WC(0001) interface and formation energies of substitutional Zr^4+^. (**a**) Schematic of the lowest energy interface structure by determined DFT calculations. (**b**) Plot of the Zr segregation energies for each position marked in (**a**). The formation energies are averaged over Al site or W sites on the respective {0001} planes.

Before calculations of Zr segregation, the most stable atomic structure of the pristine interface is determined by rigid body translations of the adjacent WC and Al_2_O_3_ slabs parallel to the interface place followed by structural optimization (see Fig. 4a). Based on this interface atomic structure determined theoretically, segregation behavior of Zr is investigated by substituting a Zr^4+^ ion at various cation sites around the interface. Figure 4b shows formation energies of substitutional Zr^4+^ around the interface. It is noted that, when Zr^4+^ replaces Al^3+^, an Al^3+^ vacancy is considered as a charge compensating defect. The formation energies are averaged over Al sites or W sites on the respective {0001} planes around the interface (Fig. 4a). It can be seen that Zr^4+^ substitution is extremely stable at the outermost W sites at the interface (denoted as “e”), as compared with that inside Al_2_O_3_ and WC grains, indicating a strong tendency of segregation of doped Zr atoms at the interface. This is in reasonable agreement with the observed Zr segregation at the interface in the HAADF image (Fig. 3a). Such a segregation behavior can also be understood from the fact that ZrO_2_ cannot dissolve into both Al_2_O_3_ and WC.

**Figure 5:**
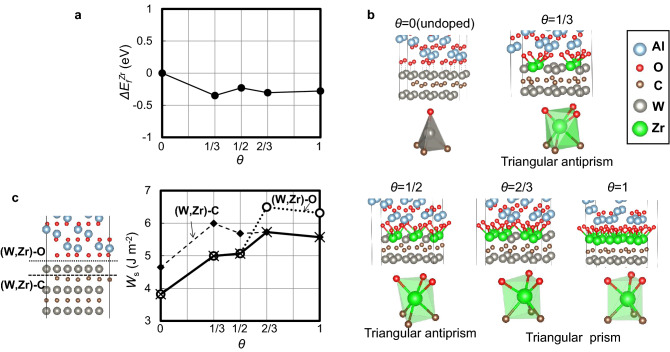
Changes in interface stability, adhesion strength, and atomic structures near the interface due to Zr segregation. (**a**) Formation energies of substitutional Zr^4+^ per defect at the interface layer as a function of Zr^4+^ occupancy *θ*. (**b**) Atomic structures of the interface layer between Al_2_O_3_ and WC. (**c**) Works of separation at the interface layer as a function of *θ*.

Since it is most likely from the STEM observation that a number of Zr atoms can simultaneously segregate at W sites of the interface layer, further DFT calculations for more intense Zr segregation are performed. In this case, some of six W sites at the interface layer in the slab model are replaced by Zr atoms so as to make substitutional Zr atoms located equally separated from one another (see also Supplementary Fig. 1). Figure 5a displays calculated formation energies of substitutional Zr per defect at the interface layer (${\Delta E}_{f}^{Zr}$) as a function of Zr occupancy ($\theta$). In all cases, ${\Delta E}_{f}^{Zr}$ shows negative values over the entire $\theta$ range. This indicates spontaneous Zr segregation at the interface layer, and intense interfacial Zr segregation can stabilize the interface.

Figure 5b represents calculated atomic structures around the interface layer between Al_2_O_3_ and WC. Before Zr segregation, W atoms at the interface layer are coordinated by four atoms (three C atoms of the WC side and one O atom of the Al_2_O_3_ side), and are located around the center of the tetrahedron. It can be seen, however, that the more intense Zr segregation induces interfacial structural changes. The original tetrahedral structure unit of the interface (*θ* = 0) can be transformed into triangular antiprisms or prisms with rising $\theta$. This can be attained by relative translations of Al_2_O_3_ and WC grains. This can be understood from the fact that Zr atoms favor sixfold or eightfold coordinations with carbon and oxygen atoms, respectively, as found in bulk ZrC and ZrO_2_. It is most likely that Zr atoms are considerably stabilized by sixfold coordinated environments in the interface layer.

**Figure 6:**
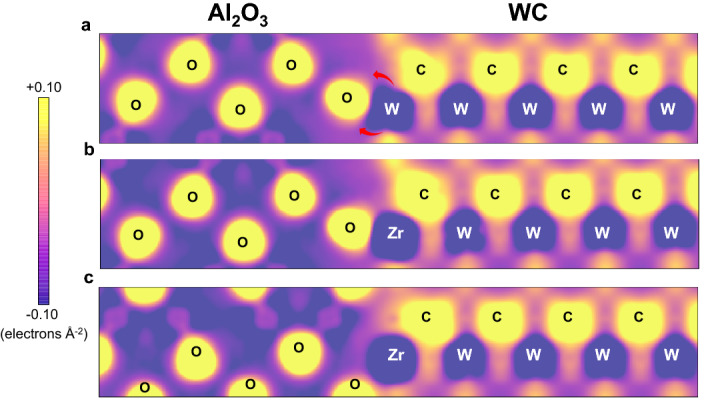
Differential electron density maps for the interfaces between Al_2_O_3_ and WC. (**a**) The most stable configuration of Al_2_O_3_ (0001)/WC (0001). (**b**) Zr substitution of W at the interface before structure optimization. (**c**) Zr substitution of W at the interface after structural optimization. These are drawn on the (10$\stackrel{-}{1}$0) plane.

As shown above, the interface between Al_2_O_3_ and WC can undertake structural changes and by intense Zr segregation. In order to investigate an electronic origin of the interfacial structural transition, electron density distributions around the interface are analyzed. Figure 6 shows differential electron density maps around the interfaces. These maps are drawn by subtracting electron densities of superimposed isolated atoms from the calculated electron densities of the interfaces, so that they represent charge transfer upon the interface formation. In the case without Zr segregation (Fig. 6a), it can be seen that electrons of the interfacial W atom tend to move toward their surrounding C and O atoms. However, the electron density of the C atom bonded to the interfacial W atom looks like a pentagon while that of the O atom has a more spherical shape. This indicates that the interfacial W atom is covalently bonded to the C atom across the interface while that also has an ionic nature of bonding with the surrounding O atom. When Zr atoms are just introduced at the interfacial W sites (Fig. 6b), it seems that electrons are also accumulated around not only O but also C in the surroundings and yet the electron density distribution of the C atom bonded to the Zr atoms is considerably distorted as compared with that before segregation (Fig. 6a). Such a distorted electron density of C is readily recovered via the structural transition (Fig. 6c). Since ionic bonds are not directional, their shear distortions do not suffer energy expenses so much as those of directional covalent bonds. Therefore, the structural changes can take place by relative translation of Al_2_O_3_ and WC across the ionic interfacial Zr-O bonds, stabilizing the interfacial Zr-C bond simultaneously. Such chemical bonding characteristics at the interface may realize its structural transition due to Zr segregation and also the resultant interfacial stability.

It is also found that Zr segregation accompanying the interfacial structural changes remarkably influences the interfacial strength. Figure 5c displays calculated works of separation (*W*_*s*_) at the interface layer as a function of $\theta$. *W*_*s*_ is obtained from total-energy differences per area between the interface and the two surfaces. In this case, two kinds of cleavage planes are considered: those between W(Zr) and O (denoted as “(W,Zr)-O”) and between W(Zr) and C (denoted as “(W,Zr)-C”). It can be seen that *W*_*s*_ for (W,Zr)-O tends to increase with increasing $\theta$ and yet becomes a maximum at *θ* = 2/3. The similar profile *W*_*s*_ is also observed for (W,Zr)-C. Since interfacial fracture should occur along planes with weaker strength, the final interface strength should follow smaller *W*_*s*_ values as displayed by the solid line in Fig. 5c. It is worth mentioning that *W*_*s*_ reaches a maximum at *θ* = 2/3, which is by 1.5 times larger than that without segregation. Therefore, Zr segregation indeed reinforces the interface between Al_2_O_3_ and WC. Formation of the interfacial structure units with increased coordination numbers of Zr is the underlying mechanism of the interfacial strengthening. Improved interfacial strength by Zr segregation as well as the excellent thermodynamic stability (see Fig. 5) should be responsible for overall mechanical strength of the Al_2_O_3_-WC composites, because higher bending strength should be closely related to fewer fracture origins in the microstructure^36,37^. As a final note, it is confirmed that ZrO_2_ addition realizes doubled life time and cutting speeds of Al_2_O_3_-WC, as compared to conventional SiC-whisker reinforced Al_2_O_3_, which proves industrial superiority of Al_2_O_3_-WC-ZrO_2_ composites developed in the present study (see Supplementary Fig. 8).

## Conclusion

In conclusion, we have achieved remarkable improvement of mechanical properties of Al_2_O_3_-WC composites via ZrO_2_ addition, which is a candidate material for cutting tool application. It was found that only Zr addition at a small amount of about 5 vol% is required for the mechanical property improvement and more ZrO_2_ addition do not contribute to further increase in mechanical strength. STEM observations demonstrated the single atomic layer segregation of Zr between Al_2_O_3_ and WC. Therefore, the atomic segregation of Zr atoms at the interface is remarkably effective to increase the strength of the composites. Energetically favorable Zr segregation just at the interface was also confirmed from DFT calculations. In addition, it was theoretically demonstrated that Zr segregation can improve interfacial strength significantly, which corresponds to the observed improvement of mechanical properties of the composites. An optimum choice of additional elements can realize unexpected improvement of superhard materials.

## Supplementary information

Supplementary Figures.

## Data Availability

The data that support the findings of this study are available from the corresponding authors upon reasonable request.
