# Supplementary material for: Advanced superhard composite materials with extremely improved mechanical strength by interfacial segregation of dilute dopants
Source: Sci Rep. 2020 Dec 3;10:21008. doi: 10.1038/s41598-020-78064-0 (PMC7712878; doi:10.1038/s41598-020-78064-0)
Supplement: Supplementary file 1 — Supplementary Figures. [file 41598_2020_78064_MOESM1_ESM.docx]

**Supplementary Information**

**Advanced superhard composite materials with extremely improved mechanical strength by interfacial segregation of dilute dopants**

Tomohiro Nishi^1,2*^, Katsuyuki Matsunaga^1,3*^, Takeshi Mitsuoka^2^, Yasuyuki Okimura^2^ and Yusuke Katsu^2^

^1^Department of Materials Physics, Nagoya University, Furo-cho, Chikusa-ku, Nagoya 464-8603, Japan.

^2^ NGK spark plug co., LTD., Iwasaki, Komaki-shi, Aichi 485-8510, Japan

^3^Nanostructures Research Laboratory, Japan Fine Ceramics Center, 2-4-1, Mutsuno, Atsuta-ku, Nagoya 456-8587, Japan.

*Corresponding authors;

E-mails: tom-nishi@mg.ngkntk.co.jp (T.N.), kmatsunaga@nagoya-u.jp (K.M)


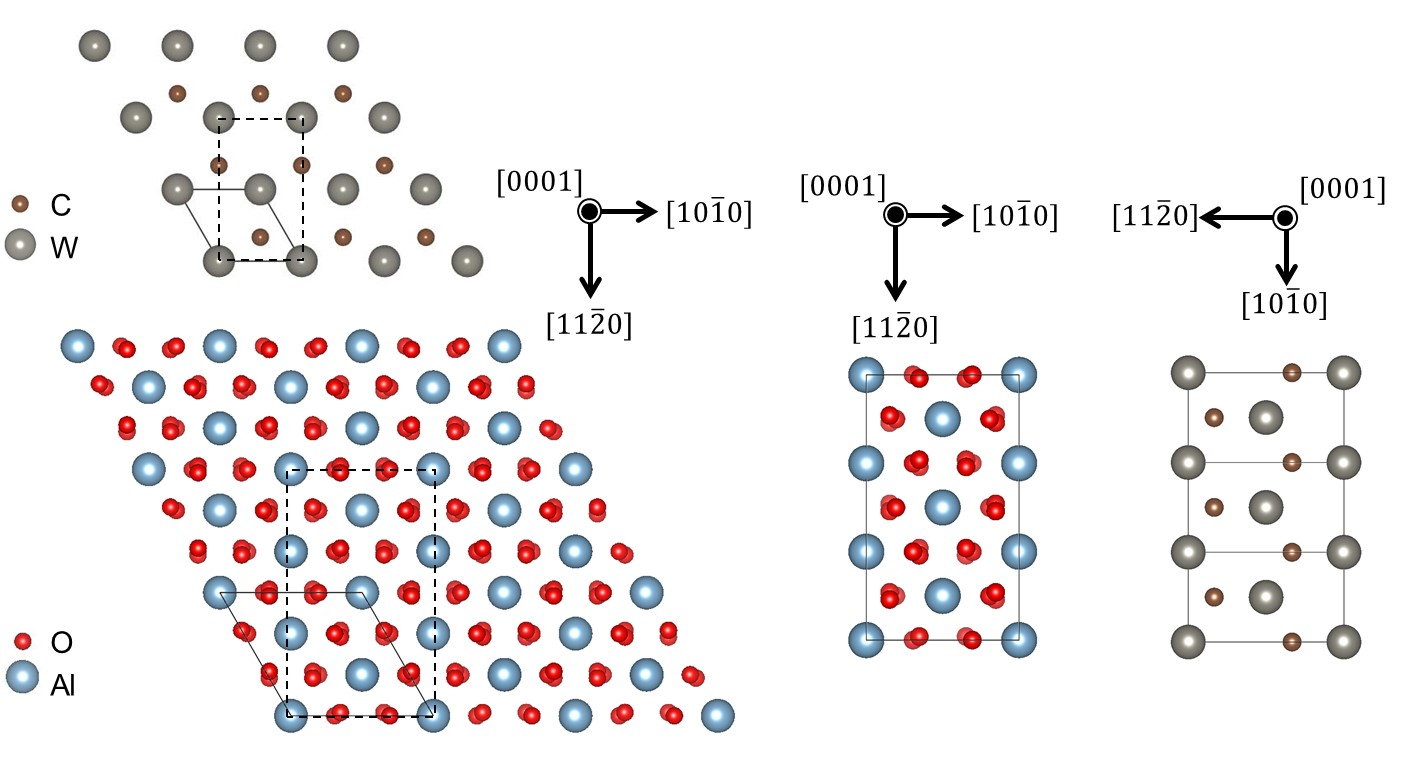


**Supplementary Figure 1 | Slab models projected on the interface plane used for DFT calculations.** A minimum unit of WC(0001) toward [10$\bar{1}$0]WC was extended by three times so as to make the lattice misfit minimized. A lattice misfit of the extended slab model toward the [11$\bar{2}$0]Al_2_O_3_ direction (the [10$\bar{1}$0]WC direction) minimized from 65% to 3.9%.


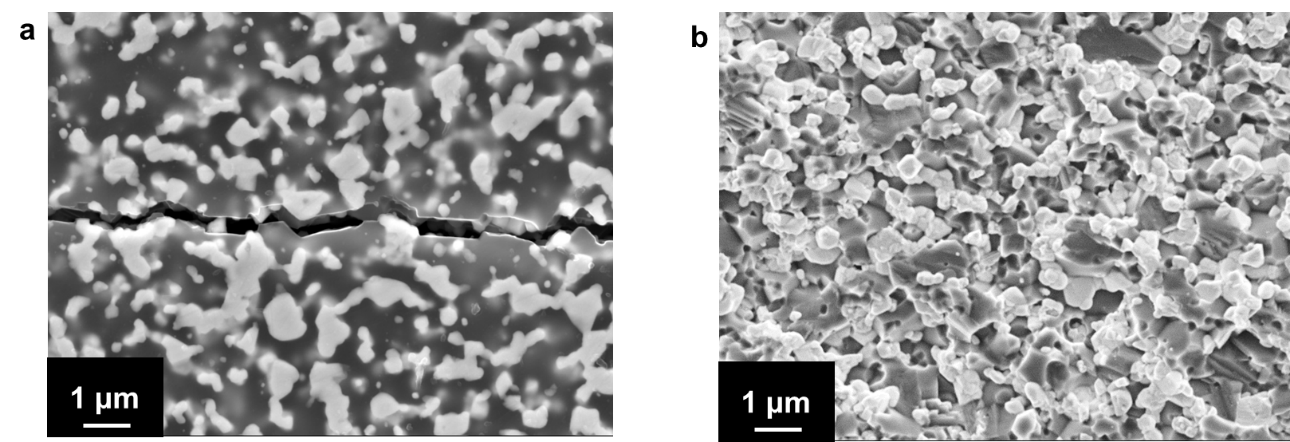


**Supplementary Figure 2 | FE-SEM images of Vickers indentation crack extension path and fracture surface of Al_2_O_3_-WC composites.** (a) Vickers indentation crack extension path and (b) fracture surface of Al_2_O_3_-45WC composite.


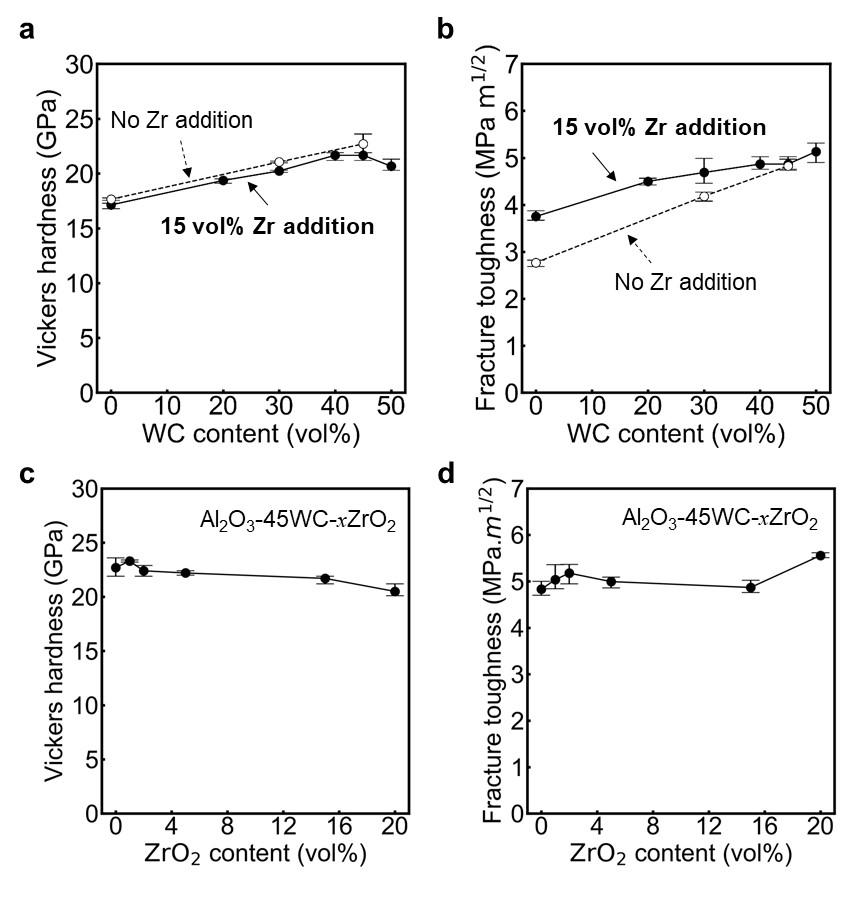


**Supplementary Figure 3 | Mechanical properties of Al_2_O_3_-WC composites.** (a) Vickers hardness and (b) fracture toughness against WC content for Al_2_O_3_-WC-15ZrO_2_ composites. (c) Vickers hardness and (d) fracture toughness against ZrO_2_ content *x* for Al_2_O_3_-45WC-*x*ZrO_2_ composites.


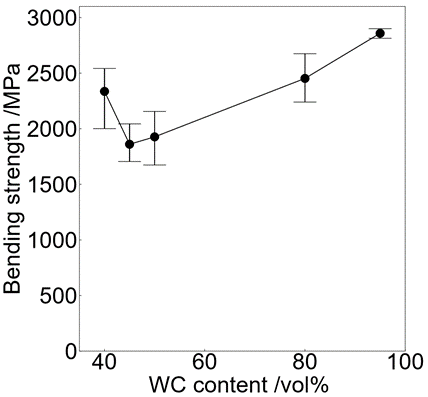


**Supplementary Figure 4 | Bending strength of Al_2_O_3_-WC-15ZrO_2_ composites.**


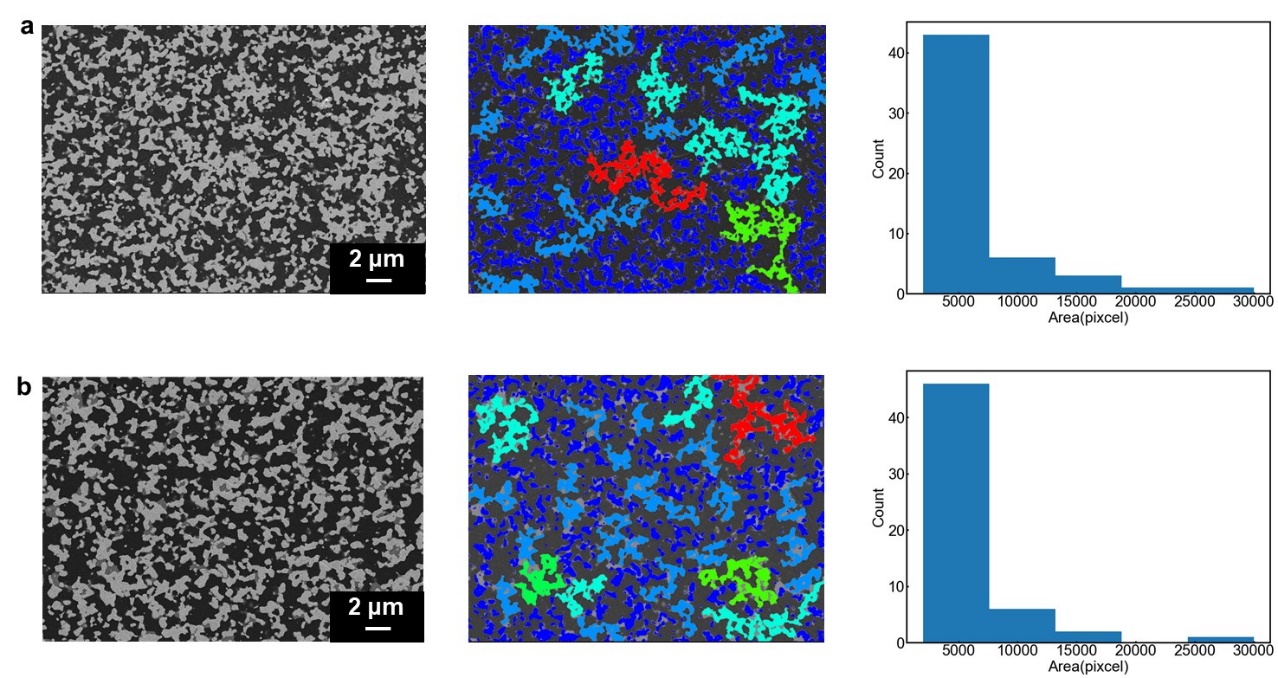


**Supplementary Figure 5 | SEM image and microstructural analysis of the Al_2_O_3_-WC composites.** (a) SEM images and histograms of the size distributions of interconnected skeletons of WC grains of Al_2_O_3_-45WC, (b) those of Al_2_O_3_-45WC-5ZrO_2_ composites. In these SEM images, brighter gray areas indicate WC grains in the microstructures.


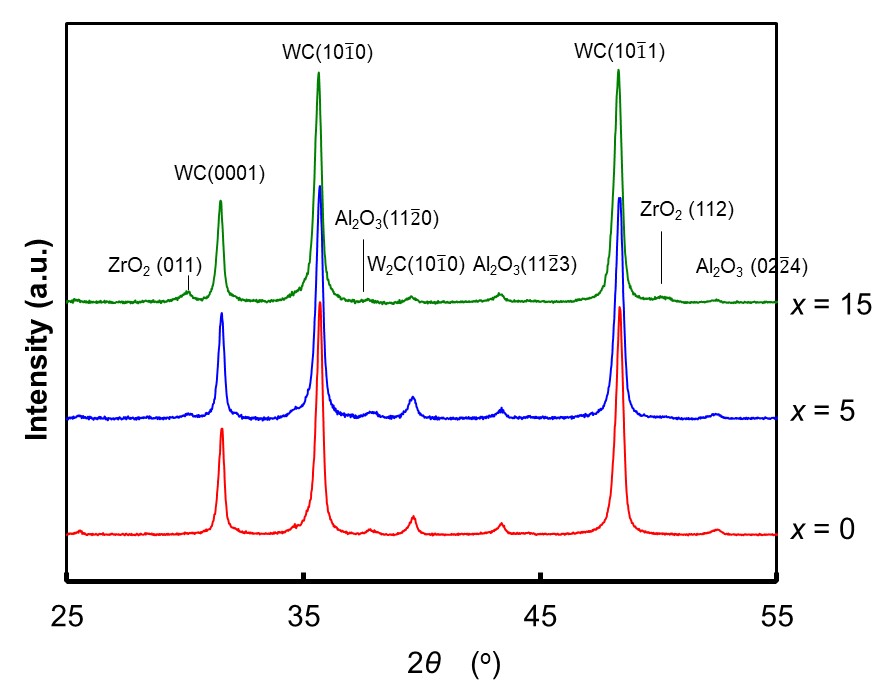


**Supplementary Figure 6 | XRD patterns of the Al_2_O_3_-WC composites (Al_2_O_3_-45WC-*x*ZrO_2_ composites with *x* = 0, 5, 15).**


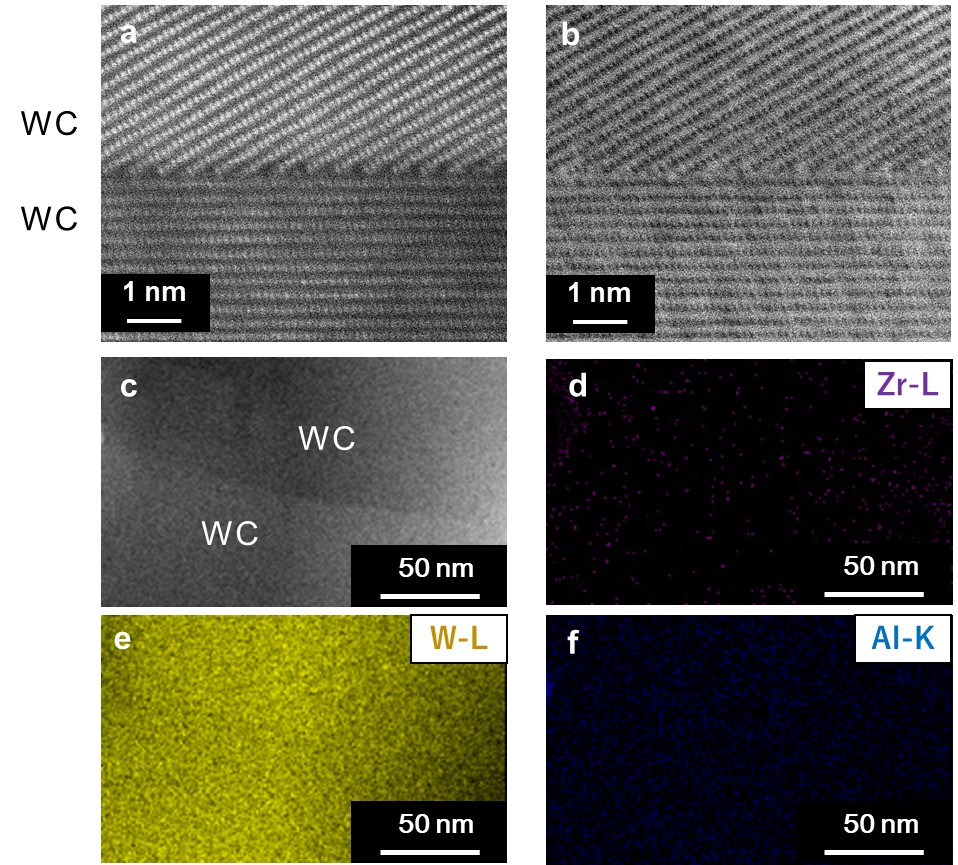


**Supplementary Figure 7 | HAADF and ABF STEM images and STEM-EDS for specimens of Al_2_O_3_-45WC-5ZrO_2_.** (a) HAADF-STEM image and (b) ABF-STEM image for a WC grain boundary of Al_2_O_3_-45WC-5ZrO_2_. (c)-(f) STEM image of a WC grain boundary and its corresponding element mapping images.


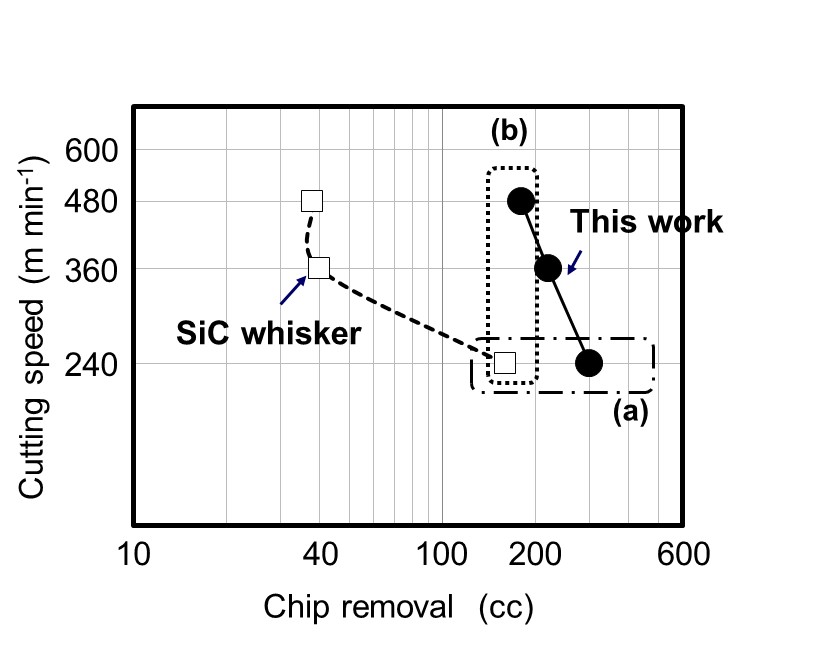


**Supplementary Figure 8 | Cutting performance. Cutting test results of Inconel 718 using the present CMCs and conventional SiC whisker ceramics.** The chip removal on the horizontal axis indicates the cutting removal amount until the tool life, and is defined as the tool life. The developed product showed twice longer tool life (cutting removal) at 240 m min^-1^, the cutting speed range of whisker type ceramics (a). Moreover, the tool life of the developed product at 480 m min^-1^ which is a double of the current cutting speed corresponds to the tool life of 240 m min^-1^ of whisker ceramics (b).
